# Efficacy, safety, and tolerability of dolutegravir-based ART regimen in Durban, South Africa: a cohort study

**DOI:** 10.1186/s12879-024-09202-6

**Published:** 2024-03-21

**Authors:** Nivriti Hurbans, Panjasaram Naidoo

**Affiliations:** 1 Discipline of Pharmaceutical Sciences, School of Health Sciences, College of Health Science, University of KwaZulu-Natal, Westville, Durban, 4001, South Africa; 2 South African Medical Research Council, HIV and Other Infectious Diseases Research Unit, Durban, South Africa

**Keywords:** Dolutegravir, Efficacy, Safety, Tolerability, HIV/AIDs, Integrase strand transfer inhibitors

## Abstract

**Background:**

Dolutegravir is an integrase strand transfer inhibitor that has been recommended for use in first-line antiretroviral regimens by the World Health Organisation and is currently being rolled out globally. There has been safety concerns with dolutegravir which has caused concern about its use in the general population. Dolutegravir first-line regimens have been used in South Africa since early 2020. Therefore, the aim of the present study was to assess the efficacy, safety, and tolerability of first-line dolutegravir-based antiretrovirals amongst adults living with HIV in Durban, South Africa.

**Methods:**

This was a mixed-methods study, which comprised a cross-sectional survey and longitudinal retrospective follow-up of medical records. The study was conducted between October 2020 and January 2022. Data were described using descriptive and summary statistics. Bivariate logistic regression was applied to socio-demographic and clinical variables and crude odds ratios with a 95% confidence interval was calculated. Pearson chi-square tests, paired sample T-tests, and cross-tabulations were performed on selected variables.

**Results:**

A total of 461 participants were enrolled in the study. There was a significant change in immunological outcomes (*p* < 0.001) after dolutegravir initiation. Furthermore, an assessment of laboratory parameters showed that there was a significant decrease in cholesterol (*p* < 0.001) and increase in creatinine (*p* < 0.001) levels. Increased weight was shown by 60.7% of the participants but was not associated with age, gender, CD4 counts, and previous antiretroviral usage. The study found that 43.6% of the participants experienced at least one side-effect. A total of 21.6% and 23.2% of the participants experienced neuropsychiatric and central nervous system side-effects, respectively. In the bivariate analyses, only gender was shown to be associated with side-effects, and only 1.7% of the participants discontinued the study due to side-effects.

**Conclusion:**

Our results suggest that dolutegravir is effective, safe, and well tolerated in the study population.

## Introduction

Human immunodeficiency virus (HIV) is a global health concern of unparalleled magnitude. Since the virus can rapidly mutate and develop resistance to available antiretroviral treatment (ART), there is a need to continuously develop new and effective drugs that are well tolerated [1]. Conventional ART consists of three drugs from at least two different classes. This type of treatment is referred to as highly active antiretroviral therapy (HAART) [2]. HAART has revolutionised HIV treatment by decreasing the number of acquired immunodeficiency syndrome (AIDS)-related deaths and opportunistic infections [3, 4]. This success is being overshadowed by the increasing rate of pretreatment drug resistance to non-nucleoside reverse transcriptase inhibitors (NNRTIs) [5]. The World Health Organisation (WHO) has encouraged countries that have > 10% pretreatment resistance to NNRTIs to consider switching to alternative antiretroviral (ARV) drugs [6, 7]. Recent reports show that South Africa (SA) has a 23.6% pretreatment resistance to NNRTIs [8].

Integrase strand transfer inhibitors (INSTIs) are the latest class of ART drugs that have been developed to target HIV enzymes [9] and are recommended for the treatment of ART-experienced and ART-naïve patients [10]. Dolutegravir (DTG), a second generation INSTI, has demonstrated both high efficacy and a high barrier to resistance [11]. However, the choice of nucleoside reverse transcriptase inhibitors (NRTIs)to be used with DTG must be carefully selected, as pretreatment drug resistance to NRTIs could compromise the efficacious performance of the DTG-based regimen. There is also the possibility that people living with HIV/AIDS (PLWHA) that have virological failure and are resistant to NRTIs may be switched to DTG-based ART [12].

Whilst DTG has shown efficacy in clinical trials, negligent implementation of the SA ART guidelines could result in increased resistance and morbidity amongst ART users [11]. Moreover, in SA, the use of DTG together with NRTIs could lead to the emergence of NRTI mutations, and resultant DTG monotherapy if there are delays in addressing viremia [13]. Furthermore, ART programmes may not implement viral load (VL) testing when transitioning individuals to DTG-based first-line ART. Switching people on ART from tenofovir + lamivudine + efavirenz to tenofovir + lamivudine + DTG in the absence of viral testing may place them at a greater risk of DTG HIV drug resistance [11].

DTG was introduced into the SA ART regimen in early 2020 as a first-line medicine in the adult population. The National ART treatment guidelines were updated to replace the tenofovir + emtricitabine/lamivudine + efavirenz/nevirapine regimen with tenofovir + emtricitabine/lamivudine + DTG. This new regimen was applicable to all adults who: were newly diagnosed, had undetectable VLs (< 50 copies/ml) and were being switched to DTG-based ART, experienced side-effects with efavirenz and those that preferred to use DTG after receiving all necessary information. A fixed dose combination of tenofovir 300 mg + lamivudine 300 mg + DTG 50 mg and a separate single formulation of 50 mg DTG is available at all government healthcare facilities nationally [14].

PLWHA have a lifelong dependency on ART. Some earlier studies have shown concern for the use of DTG outside of clinical trials due to increased discontinuation rates owing to neuropsychological, psychiatric and gastrointestinal side-effects (SEs) [15, 16]. Since DTG can revolutionise HIV treatment and is the current first-line treatment choice, with millions of PLWHA anticipated to be using this drug, especially in low- and middle-income countries, it is important that more data are collected from the general population to support its safety and efficacy when used as per the labelled instructions.

SA has been affected most relentlessly by HIV/AIDS and has accounted for more than half of AIDS related deaths globally since 1998 [17]. Consequently, SA has the biggest ART program worldwide [18, 19]. Therefore, a well-tolerated, robust and economical first-line regimen with a high genetic barrier to resistance would be advantageous. Whilst DTG is anticipated to increase rates of viral suppression as a first-line ART drug, concerns such as poor adherence and stock outs may lead to the emergence and transmission of drug-resistant HIV [11]. KwaZulu-Natal has the highest prevalence of HIV in SA. Therefore this study aimed to report on the safety, efficacy, and tolerability of DTG-based ART in a population of PLWHA in the eThekwini Metropolitan area of Durban, SA.

## Method

### Study design and research setting

This was a mixed-method study, which comprised a cross-sectional survey questionnaire and a longitudinal retrospective follow-up of medical records. Quantitative and qualitative data was collected. The study sites were three public health care facilities in the province of KwaZulu-Natal comprising two regional hospitals designated as facilities A and B and one primary health care clinic designated as facility C. All facilities were within the eThekwini Metropolitan area of Durban and were selected based on convenience sampling where each facility had to have a specialised HIV clinic, complete at least 20 to 30 ARV initiations per month, and maintain a separate filing area for ARV patient medical records.

### Study population and sample size

The study population consisted of all PLWHA that presented at the selected health facilities. To be eligible for study participation, all PLWHA had to be 18 years or older and on a first-line DTG-based ART regimen for at least 4 to 8 months at the time of enrolment into the study, and willing to provide written informed consent. A sample size of 350 participants was estimated using Stata V13.1, based on the ability to estimate the proportion of participants that experience SEs. In order to accommodate for dropouts from the study a 15% loss to follow-up was determined and included in the sample size calculation giving a minimum sample size of 415 participants.

### Data collection tools and procedures

The data collection tools included an anonymous, coded, self-administered questionnaire that contained open and closed-ended questions and a clinical file review sheet. The questionnaire was used to collect information on socio-demographics, adherence, and SEs experienced. All questionnaires were made available in English and isiZulu. The clinical file review sheet was used to collect data on clinical investigations and clinical and counselling notes. Clinical investigations were the investigations conducted and clinical notes were records that the treating clinician recorded in the medical records.

Study participants were recruited and enrolled from the waiting areas of the health facilities. A pre-screening process was implemented using the eligibility criteria. Eligible participants willing to participate in the study were given an information sheet detailing the study and thereafter were required to provide written informed consent. A self-administered questionnaire was used for this phase of the study (cross sectional). The medical files of these participants were used for the retrospective study phase. Research assistants conversant in both English and Isizulu assisted with the distribution of the questionnaires and explanation of any information that was not clear to the participants. Study recruitment and enrolment occurred between October 2020 and February 2021. Medical records were reviewed between October 2020 and January 2022 for the following information: VLs, CD4 counts, clinical staging, prior ART regimen usage, haemoglobin, creatine, cholesterol, alanine transaminase, SEs and participant weight. Study recruitment occurred during the COVID-19 pandemic, however this did not impact data collection. All necessary social distancing measures were observed during this period. The health care facilities further managed patient bookings and social distancing measures.

Efficacy was determined based on the assessment of changes in VL and CD4 cell counts and the level of adherence. Safety was determined based on the assessment of SEs and changes in laboratory parameters. Tolerability was determined based on the discontinuations from DTG due to SEs. All data from the medical records were evaluated from DTG initiation and for up to 12 months thereafter if the participant was still on DTG-based ART. The blood analysis formed part of an annual assessment. Any missing data was excluded from the analysis. Data collection occurred periodically after the participant was enrolled into the study and continued until 12 months post DTG-based ART initiation. Baseline results were the data available at the time of DTG initiation. A participant was deemed as lost to follow-up if they missed a clinic appointment by at least 3 months from the last scheduled date, and no further data collection occurred. Data collected until the participant was lost to follow-up was included in the study analysis.

### Data analysis

Data were captured onto REDCap and exported into Statistical Package for Social Sciences Version 28 for further analyses. Data were described using descriptive and summary statistics, and categorical variables were described using numbers and percentages. Bivariate logistic regression was applied to demographic and clinical variables and crude odds ratios with a 95% confidence interval were calculated to determine whether there were any associations between dependent and independent variables. Pearson chi square-tests, paired T-tests and cross-tabulations were performed on selected variables.

## Results

A total of 461 participants consented to participate and were enrolled in the study. These participants completed the questionnaire, and all 461 medical records were reviewed for further data collection.

### Socio-demographic characteristics

The socio-demographic characteristics of the study participants are summarised in Table 1.

**Table 1:** Socio-demographic characteristics of study participants (*N* = 461)

Variable	Frequency	Proportion (%)
**Health Facility**		
A B C	232 123 106	50.3 26.7 23.0
**Age Group (years)**		
18 − 30 31 − 50 > 50	33 268 160	7.2 58.1 34.7
**Gender**		
Male Female	227 234	49.2 50.8
**Marital Status**		
Single Married Living with partner	275 122 64	59.6 26.5 13.9
**Educational Level**		
No schooling Primary school Secondary school College or University	11 106 311 33	2.3 23.0 67.5 7.2
**Residential area**		
City/town Rural/farm Township	123 55 283	26.7 11.9 61.4
**Employment Status**		
Self-employed Formal employment Unemployed Missing	48 191 220 2	10.4 41.4 47.7 0.5

### Baseline characteristics

The baseline characteristics of the study participants are shown in Table 2. All 461 participant records were used, however baseline WHO clinical staging, VLs and CD4 counts were not available for all participants. The number of observations used for each variable are documented in each of the data tables. More than 95% of the study population switched to the DTG-based regimen. The study did not evaluate the length of time that the participant was on the prior regimen. The clinical notes were used to determine whether the participant had ongoing SEs at the time of DTG initiation. Previously ongoing SEs, unrelated to DTG-based ART usage were not noted as SEs for this study.

**Table 2:** Baseline characteristics of study participants [n is variable depending on availability of data]

Variable	Frequency	Proportion (%)
**ART Usage** (***n*** = **461)**		
ART-experienced ART-naïve	431 30	93.5 6.5
**WHO Clinical Staging** (***n*** = **453)**		
1 2 3	382 64 7	84.3 14.1 1.5
**VL (copies/ml)** (***n*** = **430)**		
< 50 50 − 999 ≥ 1000	397 27 6	92.3 6.3 1.4
**CD4 (cells/mm**^**3)**^(***n*** = **403**)		
< 200 200 − 350 351 − 500 > 500	28 47 90 238	6.9 11.7 22.3 59.1
**Previous ART Regimen** (***n*** = **431**)		
Tenofovir/Emtricitabine/Efavirenz Other	411 20	95.4 4.6

### Efficacy of DTG-based ART

The VL, CD4 counts and adherence were used to describe efficacy. VLs < 50 copies/ml, CD4 counts > 500 cells/mm^3^ and self-reported adherence of ≥ 95% were considered the optimal measurements. Changes in VL and CD4 counts from baseline was determined and were subjected to statistical analysis using a paired T-test.

### Virological and immunological outcomes

The changes in virological and immunological outcomes of study participants taking DTG-based ART are presented in Table 3. The results presented are for participants for whom baseline and follow-up data were available in the medical records. There were no statistically significant differences in VLs in the proportion of participants on ART at baseline and after DTG-based ART (*p* = 0.138). VLs remained unchanged in 345 (84.5%) of the participants who had VL < 50 copies/ml at baseline. Increased VLs were shown in participants as follows: 27 (6.6%) from < 50 copies/ml to 50 − 999 copies/ml and 4 (1.0%) from < 50 copies/ml to ≥ 1000 copies/ml. Decreased VLs from 50 − 999 copies/ml to < 50 copies/ml, from ≥ 1000 copies/ml to < 50 copies/ml and from ≥ 1000 copies/ml to 50 − 999 copies/ml were shown by 27 (6.6%), 4 (1.0%), and 1 (0.2%) participant, respectively.

**Table 3:** Virological and immunological outcomes in participants taking DTG-based ART (n is variable depending on availability of data)

Variable	Baseline Frequency (Proportion %)	After DTG-based ART Frequency (Proportion %)	T-test (p)
**VL (copies/ml)** (***n*** = **408)**			
< 50 50 − 999 ≥ 1000 Mean	376 (92.2) 27 (6.6) 5 (1.2) 337.41 ± 3365.26	375 (91.9) 29 (7.1) 43 (1.0) 88.54 ± 325.68	0.138
**CD4 (cells/mm**^**3**^)(***n*** = **344)** < 200 200 − 350 351 − 500 > 500 Mean	19 (5.5) 39 (11.3) 73 (21.2) 213 (61.9) 599.49 ± 261.36	6 (1.7) 38 (11.1) 47 (13.7) 252 (73.3) 681.67 ± 290.40	< 0.001

There were statistically significant differences in CD4 counts in the proportion of participants on ART at baseline and after DTG-based ART (*p* < 0.001). CD4 counts remained unchanged in 238 (69.2%) participants. Decreased CD4 counts were shown as follows: 15 (4.4%) from > 500 cells/mm^3^ to 351 − 500 cells/mm^3^, 3 (0.9%) from > 500 cells/mm^3^ to 200 − 350 cell/mm^3^ and 9 (2.6%) from 351 − 500 cells/mm3 to 200 − 350 cells/mm3. Increased CD4 counts were seen in 79 (23.0%) participants.

### Adherence to DTG-based ART

Table 4 summarises self-reported adherence. A total of 69 (15.0%) of the study participants reported having missed clinic appointments and 19.1% reported missing between 1 and 5 doses over a period of 6 months. Reasons for missed clinic appointments included being busy (*n* = 43), feeling unwell (*n* = 6), and other (*n* = 20). The reasons cited under “other” by study participants included forgotten dates, travelling away from home, and the restrictions on movements during the COVID-19 pandemic. The proportion of participants that shared or borrowed ART was 2.0% and 2.2%, respectively. Pearson chi-squared analyses, shown in Table 2, which were performed to determine any significant relationships between missed clinic appointments, forgotten doses, ART sharing, ART borrowing, gender, and prior ART usage, showed that there was a significant relationship between missed clinic appointments and ART sharing (*p* < 0.001).

**Table 4:** Measurement of adherence to DTG-based ART by self-report

Variable	Frequency	Proportion (%)	Pearson chi-squared test (p)
**Missed clinic appointments**			-
Yes No	69 392	15.0 85.0	
**Forgotten doses**			> 0.05
None Between 1 − 5 times Between 6 − 10 times > 10 times	367 88 4 2	79.6 19.1 0.9 0.4	
**ART sharing**			> 0.05
Yes No	9 452	2.0 98.0	
**ART borrowing**			< 0.001
Yes No	10 451	2.2 97.8	

### Safety of DTG-based ART

Safety considerations was based on the observed and reported SEs and changes in laboratory parameters. Optimal safety was determined by the severity of SEs that led to treatment related discontinuations and was measured by < 5% treatment discontinuations due to SEs. Changes in laboratory parameters from baseline was determined and were subjected to statistical analysis using a paired T-test. The normal values for each of the parameters assessed are described in Table 5, with decreased haemoglobin and creatinine and increased cholesterol and alanine transaminase being the markers of safety determination.

**Table 5:** Changes in laboratory parameters after taking DTG-based ART

Variable	Baseline Frequency (Proportion %)	After DTG-based ART Frequency (Proportion %)	T-test (p)
**Haemoglobin** (***n*** = **204**)			
Normal (12 − 15 g/dl) Low (< 12 g/dl) Mean	156 (76.5) 48 (23.5) 12.96 ± 1.49	156 (76.5) 48 (23.5) 12.92 ± 1.45	0.555
**Cholesterol** (***n*** = **169)**			
Normal (< 5 mmol/L) Elevated (≥ 5 mmol/L) Mean	121 (71.6) 48 (28.4) 4.5 ± 0.96	145 (85.8) 24 (14.2) 4.2 ± 1.02	< 0.001
**Alanine transaminase** (***n*** = **197)**			
Normal (7 − 35 U/L) Elevated (> 35 U/L) Mean	162 (82.2) 35 (17.8) 27.28 ± 14.69	173 (87.8) 24 (12.2) 25.33 ± 16.76	0.129
**Creatinine** (***n*** = **383)**			
Normal (49 − 90 µmol/L) Elevated (> 90 µmol/L) Low (< 49 µmol/L) Mean	320 (85.6) 40 (10.4) 23 (6.0) 71.73 ± 16.73	254 (66.3) 121 (31.6) 8 (2.1) 84.13 ± 19.29	< 0.001

### Side-effects (SEs)

A total of 453 SEs were experienced by 201 (43.6%) participants. The SEs experienced are summarised in Table 6. Neuropsychiatric (NP) and central nervous system (CNS) SEs accounted for 98 (21.6%) and 105 (23.2%) respectively, of the total number of SEs experienced. Of the NP SEs, 92 of 98 (93.9%) cases were reported by ART-experienced participants, with insomnia being reported by 44 of the 98 participants who experienced NP SEs. Headache and dizziness were the two most commonly reported CNS SEs, while abdominal pain and nausea were the two most common gastrointestinal SEs and malaise/tiredness being the commonest musculoskeletal SE.

**Table 6:** SEs experienced by participants after taking DTG-based ART (*N* = 461)

Variable	ART Naive		ART Experienced	
	Frequency	Proportion of total (%)	Frequency	Proportion of total (%)
**Neuropsychiatric** (***n*** = **98**)				
Abnormal dreams Insomnia Depression Confusion	2 4 0 0	0.4 0.9 0 0	27 40 6 19	5.9 8.7 1.3 4.2
**Central Nervous System** (***n*** = **105)**				
Dizziness Headaches Memory loss Neuralgia	1 2 0 0	0.2 0.4 0 0	40 56 1 5	8.8 12.4 0.2 1.1
**Gastrointestinal** (***n*** = **80)**				
Nausea Vomiting Abdominal pain Constipation Diarrhoea Heartburn	1 0 2 0 1 0	0.2 0 0.4 0 0.2 0	28 4 36 3 2 3	6.2 0.9 7.9 0.7 0.4 0.7
**Dermatological** (***n*** = **70)**				
Rash Dry skin	8 0	1.8 0	59 3	13.0 0.7
**Musculoskeletal** (***n*** = **72)**				
Malaise/tiredness Arthralgia Cramps	5 0 1	1.1 0 0.2	56 7 3	12.4 1.5 0.7
**Miscellaneous** (***n*** = **28)**				
Enlargement of breasts Cough Edema Loss of appetite Facial swelling Night sweats Weight gain Weight loss	0 0 0 1 0 0 1 0	0 0 0 0.2 0 0 0.2 0	12 2 1 3 3 1 3 1	2.6 0.4 0.2 0.7 0.7 0.2 0.7 0.2

Assessments of changes in the weight of the study participants after taking DTG-based ART are presented in Table 7. It was found that 12.9% of the participants did not show any changes in weight over the study duration; however, 60.7% of participants experienced increases in weight to different extent, i.e. 32.2%, 17.2%, and 11.3% showed a 1 − 5%, 6 − 10%, and > 10% increase in weight, respectively. The Pearson chi-square test, which was applied to age, gender, CD4 counts, and previous ART usage to determine whether there was any relationship to weight gain, showed that there were no statistically significant associations relating to these variables (*p* > 0.05 in all instances).

**Table 7:** Changes in weight after taking DTG-based ART (*n* = 425)

Weight change from baseline	Frequency	Proportion (%)
No change 1 − 5% increase 6 − 10% increase > 10% increase 1 − 5% decrease 6 − 10% decrease > 10% decrease	55 137 73 48 74 33 5	12.9 32.2 17.2 11.3 17.4 7.8 1.2

### Laboratory parameters

The changes in laboratory parameters of study participants taking DTG-based ART are presented in Table 5. Using a paired T-test, we compared the effect of DTG-based ART on laboratory parameters. It was shown that only changes in cholesterol and creatinine were statistically significant (*p* < 0.001).

### Factors associated with the safety of DTG-based ART

Bivariate logistic regression was applied to demographic and clinical variables to determine factors associated with safety to DTG-based ART, summarised in Table 8. On reviewing the crude odds ratio, only gender showed statistical significance. Females in the study were 1.7 times more likely to experience a SE compared with males.

**Table 8:** Factors associated with the safety of DTG-based ART (*N* = 461)

Variable	Side Effects Experienced		Crude Odds Ratio (95% CI)
	Yes	No	
**Age Group**			
18 − 30 31 − 50 > 50	19 150 90	14 118 70	1 1.000 (0.390, 2.563) 1.004 (0.372, 2.708
**Gender**			
Male Female	141 118	86 116	1 1.747 (1.089, 2.803)
**ART Usage**			
ART experienced ART naïve	246 13	185 17	1 -
**CD4 count (cells/mm** ^**3**^ **)**			
< 200 200 − 350 351 − 500 > 500	10 28 51 134	17 18 39 105	1 0.260 (0.063, 1.076) 0.305 (0.081, 1.148) 0.287 (0.078, 1.058)
**VL (copies/ml)**			
< 50 50 − 999 ≥ 10,000	226 14 4	170 14 2	1 1.557 (0.635, 3.814) 0.403 (0.035, 4.591)
**Clinical Staging**			
1 2 3	210 39 6	172 25 1	1 0.586 (0.313, 1.097) 0.000
**Co-morbidity**			
Hypertension Diabetes Hypercholesterolemia Arthritis Tuberculosis	66 20 16 17 5	48 8 10 13 3	1.174 (0.694, 1.976) 1.582 (0.632, 3.961) 0.730 (0.285, 1.869) 1.343 (0.589, 3.060) 0.799 (0.126, 5.073)

### Tolerability

A total of 53 (11.5%) of the participants discontinued their participation in the study prior to the 12-month follow-up period. The reasons for DTG discontinuations are summarised in Table 9. Only eight participants discontinued DTG-based ART due to SEs and they presented with a wide range of neuropsychiatric, CNS, gastrointestinal, dermatological, musculoskeletal and miscellaneous SEs.

**Table 9:** Reasons for participants discontinuing DTG-based ART (*n* = 53)

Reason	Frequency	Proportion (%)
Participant transferred out to another health facilities	34	64.2
Participant lost to follow-up	10	18.9
Participant experiencing side effects	8	15.1
Type of SE: Neuropsychiatric (*n* = 6) Insomnia (*n* = 3); Depression (*n* = 1); Confusion (*n* = 2) Central Nervous System (8) Dizziness (*n* = 5); Headaches (*n* = 2); Neuralgia (*n* = 1) Gastrointestinal (7) Nausea (*n* = 1); Vomiting (*n* = 1); Abdominal pain (*n* = 4); Diarrhoea (*n* = 1) Dermatological (*n* = 2) Rash (*n* = 2) Musculoskeletal (*n* = 4) Malaise (*n* = 4) Miscellaneous (*n* = 4) Enlargement of breasts (*n* = 2); Facial swelling (*n* = 1); Weight gain (*n* = 1)		
Other (participant wanted to conceive)	1	1.8

## Discussion

It is evident from the results of this study, that DTG is an effective, safe, and tolerable drug that can be used in the first-line ART regimen. This study has contributed to the body of knowledge of information regarding the efficacy, safety, and tolerability of DTG in the SA adult population.

A comparison of the virological response after DTG initiation did not show any significant changes from baseline. A total of 31 participants showed increased VLs. Four (0.9%) of the ART-experienced participants had baseline VL counts ≥ 1000 copies/ml at the time of switching to DTG-based ART, despite the WHO recommendations that patients on an NNRTI backbone should have a VL of < 1000 copies/ml before switching to DTG-based ART [20, 21]. Nonetheless, after switching, three of these participants achieved undetectable VLs (< 50 copies/ml), whilst one participant had a VL that measured 59 copies/ml. None of these four participants discontinued DTG-based ART and only one experienced a SE in the form of a skin rash. Uncontrolled VLs may be indicative of resistance mutations [22]. The literature suggests that slow virological outcomes may be due to transmitted resistance [23].

Approximately 62% of the study participants were shown to have a CD4 count of > 500 cells/mm^3^ at baseline, with a significant improvement in immunological response after receiving DTG-based ART. According to our knowledge, there are limited literature resources regarding improvements in CD4 counts after initiating DTG-based ART, and therefore this is positive data that should be confirmed by further studies.

An adherence of ≥ 95% to ART is required to achieve and maintain viral suppression [24]. Self-reported adherence was shown to be good in the present study, with 80% of the study participants reporting 100% adherence to DTG-based ART over a 6-month period. ART sharing/borrowing remained low but could lead to the assumption that participants wanted to take ART even if they had run out of their own supply of medicines. A drawback of this observational study is that a more direct measure of adherence measurement, notably, pill counts, could not have been conducted. An indirect measure of adherence is through VL monitoring, where controlled VLs are an indicator of good adherence [25]. We did not note any significant increase in VLs after DTG-based ART initiation, which further supports our finding of good adherence. The frequency at which VLs were measured was as described by the National ART guidelines [14], and the most recent value on hand was used in the data analysis.

We categorised SEs experienced by the participants using the classification as described by Bhatti et al. [26]. Our study showed that 20% of the reported SEs experienced were categorised as NP, which is in keeping with several other studies in which NP SEs have also been frequently reported [15, 16, 27], including being a factor in treatment discontinuations [16, 28]. Other common DTG-related SEs reported in this study (dizziness, abdominal pain, weight gain, insomnia, and tiredness) have also been extensively reported in the literature [29–31]. However, other SEs that were reported by the study participants, namely, memory loss, neuralgia, constipation, heartburn, muscle cramps, cough, and night sweats, have previously not been reported in the literature. The constipation, heartburn, and muscle cramps were deemed unrelated to DTG, as these conditions were reported to have resolved spontaneously. With regard to neuralgia and memory loss as a SE with DTG-based ART, no sourced literature was able to confirm this relationship or elaborate it as a SE of DTG. However most literature cite the neuropsychiatric SEs associated with DTG [15, 16, 27] and memory loss [29–31] prevalent in HIV/AIDS patients. A cross-tabulation was done and we were able to determine that only TB positive study participants presented with night sweats and cough.

We found that a large proportion (60.7%) of participants experienced weight gain to varying extent; a cross-tabulation showed, no association was found between weight gain and age, gender, or baseline CD4 count. Increases in weight have been an anticipated outcome, with the WHO cautioning on the probability of weight increases with DTG-based regimens [32]. Reasons for weight gain could be linked to a decrease in energy usage after a decrease in VL [3, 4, 33] or an interaction between DTG and the melanocortin 4 receptor which regulates the caloric intake by regulating leptin in the CNS [3, 34]. Early on in the epidemic, weight gain was associated with an improvement in immunological response, which is consistent with findings of other studies [35, 36]. However, studies have shown that there is mounting trepidation amongst patients regarding unintended weight gain with DTG-based ART [3, 37]. The literature shows that ART-experienced people who switched from other regimens to INSTI-based ART also demonstrated increased weight gain [38]. While we did not find any association between gender or baseline CD4 count and weight gain, studies have shown that weight gain was significant in women [39, 40], and associated baseline CD4 counts [40]. Although DTG-based ART has been associated with increased morbidity and mortality due to clinical obesity [41]. Discontinuation of DTG-based ART due to weight gain was found in only one participant who had a 13.3% increase in weight.

Our finding of significantly lower cholesterol levels after DTG-based ART than those at baseline conflicts with results from some randomised trials where DTG was not shown to have caused any changes in serum lipid levels [42, 43]. All study participants were provided with lifestyle modification counselling at each visit, and this could be a possible explanation for the improvement in the lipid profiles of the study participants. Since DTG is a new addition to the first-line ART guidelines, there could have been greater emphasis placed on healthy lifestyle counselling, which was recorded in the medical records at each visit.

We observed increased creatinine levels in the study participants. These changes were anticipated and were significant. It is well documented that DTG causes increased serum creatinine levels by inhibiting the active secretion of organic cation transporter 2, thereby mimicking decreased renal function [44, 45]. Similar findings have been reported by Gutiérrez et al., who reported that approximately 15% of participants demonstrated insignificant but predictable increases in serum creatinine [45]. When assessing kidney function, measures that remain unaffected by DTG should therefore be used.

When examining factors associated with safety of DTG, in the bivariate analysis, we found that females were about 1.7 times (Crude odds ratio: 1.747, CI 1.089, 2.803) more likely to experience SEs compared with males. Age, previous ART usage, CD4 counts, VL, clinical staging, and comorbidity did not show any relationship with SEs.

In this study, we showed good tolerability. Only a small proportion of the total number of discontinuations (15%) were due to SEs, and these occurred in ART-experienced participants who were previously receiving tenofovir, emtricitabine, and efavirenz. Furthermore, these participants were females, aged 36 and above, with CD4 counts > 500 cells/mm^3^, undetectable VLs, and clinical stage 1 at baseline. Follow-up VL (*n* = 5) showed increased VL for one participant. Only three of these participants reported comorbidities (arthritis, hypertension, and asthma) and none reported missing any doses. The participants remained on the DTG-based ART for 7.5 ± 1.73 months before discontinuing treatment. Discontinuations due to DTG and INSTIs-related side effects have also been reported in the literature [16, 46]. Discontinuations due to CNS and NP SEs are also widely reported [15, 16, 47, 48]. It was also shown that DTG discontinuations occurred three times more frequently in women compared to men and in patients who were 60 years or older [16]. Some post-marketing observational studies showed that DTG was well tolerated in real-life settings but discontinued at a much higher rate than that reported in randomised clinical trials [47].

### Limitations

Due to the limited sample size, inferential statistics could not be applied to identify factors associated with increased VL. Our study could not determine the prevalence of obesity because of the inability to calculate body mass index due to the lack of data on the participants’ height from the medical records. Since the study enrolled participants who had been on DTG-based ART for 4 − 8 months, we were unable to determine whether there were any discontinuations due to safety concerns in the first few months after exposure to DTG. Due to this being an observational study, a more direct measure of adherence could not have been implemented. Furthermore, we were unable to determine whether weight changes had any impact on clinical obesity due to the lack of data from the medical records.

### Conclusion and recommendations

Since this study was conducted in the epicentre of the HIV epidemic (Durban, South Africa), these findings may be generalizable. The study showed that the use of DTG-based ART is effective, safe, and tolerable in the studied population.

Some recommendations that emanated from this study as a consideration for further studies with a larger sample size include: further investigations into whether NP SEs are related to a neurotoxic effect, reasons why females may experience more SEs than males, factors associated with increased VLs and whether changes in participant weight is related to changes in body mass index.

## Data Availability

The data used/analyzed in this paper can be obtained from the corresponding author upon reasonable request.

## Abbreviations

AIDS: Acquired immunodeficiency syndrome
ART: Antiretroviral treatment
ARV: Antiretroviral
CNS: Central nervous system
DTG: Dolutegravir
HAART: Highly active antiretroviral therapy
HIV: Human immunodeficiency virus
INSTI: Integrase strand transfer inhibitor
NNRTI: Non-nucleoside reverse transcriptase inhibitor
NP: Neuropsychiatric
NRTI: Nucleoside reverse transcriptase inhibitor
PLWHA: People living with HIV/AIDS
SA: South Africa
SE: Side-effect
VL: Viral load
WHO: World Health Organization
